# System changes to empower primary care in Alzheimer's disease detection and care

**DOI:** 10.1002/alz.71276

**Published:** 2026-04-28

**Authors:** Sharon Phares, Ian Kremer, J. K. Wall, Julie Wood, Matthew Baumgart, Sean Dickson, Matt Fickie, Tom Hubbard, Ali Jannati, Mark Monane, Caitlin Rivet, Gigi Hirsch

**Affiliations:** ^1^ Tufts Medical Center Institute for Clinical Research and Health Policy Studies Center for Biomedical System Design & NEWDIGS Tufts University School of Medicine Boston USA; ^2^ LEAD Coalition (Leaders Engaged on Alzheimer's Disease) Washington USA; ^3^ Eli Lilly and Company Indianapolis USA; ^4^ American Academy of Family Physicians (AAFP) Leawood USA; ^5^ Alzheimer's Association Chicago USA; ^6^ AHIP Washington USA; ^7^ Highmark Pittsburgh USA; ^8^ Tufts Medical Center, Institute for Clinical Research and Health Policy Studies Center for Biomedical System Design & NEWDIGS Boston USA; ^9^ Linus Health Boston USA; ^10^ C2N Diagnostics St. Louis USA; ^11^ Tufts Medical Center Boston USA

**Keywords:** access to care, Alzheimer's disease, biomarkers, biomedical health efficiency, cognitive testing, diagnostics, disease‐modifying therapy, disparities, early diagnosis, early intervention, health systems, primary care, reimbursement, system barriers, system design

## Abstract

Despite advances in disease‐modifying treatments, significant barriers in the evaluation and clinical management of people with early Alzheimer's disease (AD) remain. These barriers have been documented in scientific literature and increasingly call for primary care to play a larger role in the detection, diagnosis, treatment, and monitoring of AD. Drawing upon the work of a multistakeholder consortium, this article identifies systemic and structural barriers that hinder primary care professionals in the United States from playing a larger role. We propose solutions to these barriers and call for evolution of the U.S. healthcare system to ensure it is prepared to adapt to the rapidly progressing scientific and societal landscape and meet the growing needs of people affected by the early stages of AD.

## Highlights

- Better outcomes from emerging Alzheimer's disease therapies require system change.
- Empowering primary care is critical to early Alzheimer's disease detection and care.
- Delays in detection and diagnosis exacerbate health disparities.
- Emerging biomarkers and digital tools can accelerate detection and diagnosis.
- Payer policies must be aligned to support primary care management of Alzheimer's.

## 1 BACKGROUND

The prevalence of Alzheimer's disease (AD) exceeds the capacity of available health care infrastructure, resulting in long wait times, delayed or missed diagnoses, and disparities in access to care. This progressive neurodegenerative disease is the most common cause of dementia, accounting for 60%–80% of cases. AD severity is classified into stages 1 through 6.^1^ In stages 1 and 2, people have positive levels of proteins associated with AD, but typical cognitive assessments identify no impairment. Even though people in Stage 2 may experience subtle and overlooked signs of cognitive impairment, in this paper we will refer to people in both stages 1 and 2 as unimpaired. Stages 3 and 4 (“early symptomatic AD”) are characterized by mild cognitive impairment (MCI) in Stage 3 moving to a mild stage of dementia (Stage 4). Stages 5 and 6 indicate moderate and severe dementia, respectively. Individuals in Stages 1 and 2 have a 33%–50% chance of progressing within 5 years to the early symptomatic stage 3 (MCIs) and stage 4 (mild dementia). Our research focuses on increasing healthcare system capacity for the detection, diagnosis, treatment, and monitoring of the early stages of AD (stages 1–4).

It is now possible to delay or reduce some of the personal, societal, and economic costs of AD thanks to advances in DMTs for AD that slow disease progression. Presently, two United States Food and Drug Administration (FDA)‐approved disease‐modifying therapies (DMTs) are commercially available, and a robust Alzheimer's drug development pipeline for new therapies is underway. The benefits of current DMTs are modest, slowing progression to more severe stages by 27%–39% during 18‐month clinical trials with continued benefit out to 4 years.^2^, ^3^ Current DMTs are most effective in the early stages when cognitive impairment is mild. Some providers and patients have concluded those benefits are not worth it and have opted to not use these therapies. However, other patients and providers have reported strong positive responses. The debate on the merits of DMTs is beyond the scope of this paper, yet we contend that the U.S. health care system should be set up to enable access to DMTs for those who are eligible for their use and want to use them.

Today, initiating treatment requires a multistep clinical journey fraught with significant barriers to patient detection, diagnosis, treatment, and monitoring. These barriers constrain the healthcare system's ability to identify people who are eligible for DMTs and inform them of this treatment option before they progress too far in their disease to be eligible.

Of the current U.S. population age 65 and older, there are approximately 7 million people in the unimpaired stages.^4^ An additional 5 to 7 million are estimated to have MCI due to AD, and 7.2 million, or one in every 9 people in the United States over age 65, are estimated to have dementia caused by AD (stages 4–6).^5^ Demographic changes are expected to more than double those with MCI and nearly double the number with AD dementia by 2060.^6^ Although AD dementia has the highest prevalence among those over age 65, it also impacts those under the age of 65, with an estimated prevalence of 200,000 in the Unitede States.^7^ Younger‐onset AD risk is particularly high among people with Down syndrome, with the mean age of AD dementia onset of 54.5, about two decades earlier than those without the additional risk factor of trisomy 21.^8^

In contrast to the number of people with AD, the supply of memory specialists available falls far short, creating significant access challenges. Neurologists, geriatricians, and geriatric psychiatrists make up only about 2.5% of all U.S. physicians,^9^ and few new physicians choose to enter these specialties due to comparatively lower salaries, lack of mentorship, heavy workloads, high burnout risk, and limited career advancement opportunities.^10^ Consequently, patients referred to a specialist after cognitive symptoms are detected and a preliminary dementia diagnosis is made face long wait times or may never be seen at all. A 2019 analysis of Medicare beneficiaries found that only 36% of those who received a preliminary dementia diagnosis were seen by a specialist within 5 years.^11^ Even when a referral to a memory specialist is made, projected wait times in the coming years are expected to rise to more than 18 months.^12^ Combined with a rapidly aging population, this reliance on specialists to confirm diagnoses and prescribe DMTs is unsustainable, costly, and ultimately unnecessary given the number in the U.S. who will need early AD care.

Scientific literature has extensively documented the barriers to care for people in the early stage of AD and increasingly calls for primary care professionals (PCPs) to play a larger role in its detection, diagnosis, treatment, and monitoring. However, most research has focused on justifying greater primary care involvement without addressing the system structures underlying these barriers that make it more challenging for primary care to assume an expanded role.

## 2 STRUCTURAL BARRIERS TO AD DETECTION, DIAGNOSIS, TREATMENT, AND MONITORING

Structural barriers have left the current healthcare infrastructure insufficient to meet both the present and anticipated demand for early AD detection, diagnosis, treatment, and monitoring. Factors such as limited access, specialist shortages and long wait times, resource limitations, lack of specialized diagnostic services, and inadequate training of PCPs all contribute to missed, delayed, or inaccurate diagnoses, along with potentially missed opportunities to initiate and benefit from DMTs, consider enrollment in a clinical trial, or seek alternative options for treatment and care. These gaps further exacerbate health disparities and inequities.^13^

Detection and diagnosis of AD require multiple clinical steps, and patient attrition can occur at any point due to challenges such as long wait times, travel burdens, and out‐of‐pocket costs. As a result, access to care is often delayed and inequitable. These challenges are most present in those who are poor, non‐white, have limited English‐speaking skills, live in rural areas, or are female. Underlying causes include socioeconomic and educational disadvantages, limited healthcare access, comorbid conditions (particularly cardiovascular disease), genetic risk factors, environmental exposures, and low health or linguistic literacy.^14^, ^15^ Additionally, when cognitive assessments are conducted, Black and Hispanic patients are more likely to receive false positive results (Type I)^16^ and women are more likely to have early AD undetected, as verbal memory skills can mask symptoms of cognitive decline in standardized tests.^17^ Many individuals lack awareness or motivation to seek care and may deny or not recognize symptoms, especially those that do not significantly impact daily function.^18^ Fear, stigma, and discrimination are also common. Cognitive decline is often falsely believed to be a normal part of aging, leading people to underreport symptoms.^18^ Despite these factors, qualitative studies indicate that older adults and family members are interested in routinely being asked about memory problems to learn if they are at increased risk of AD.^19^ Furthermore, patient receptivity to cognitive testing improves when they are aware of new treatments for early AD.^20^

The shortage of memory specialists in many regions (creating “neurology deserts”) is another major source of delays. People in rural areas face specialist wait times roughly three times longer than those in urban areas.^21^ According to the Alzheimer's Association, 34% to 59% of people in the United States aged 65 and older live in areas with potential specialist shortfalls.^5^ This translates to an average wait time of 18 months for an initial specialist visit in newly suspected AD and often over 4 years for those already diagnosed.^22^ In addition to the shortage of memory specialists, lack of access to comprehensive neuropsychological testing makes differential diagnosis of AD challenging and often results in inaccurate or delayed diagnoses and a lack of personalized treatment planning.^23^ Because easy‐to‐administer diagnostic tools, while available, are not typically reimbursed, it adds to time delays in diagnosis for those living with this progressive disease and often means diagnosis is never formally made or made only after AD has progressed to more severe stages and eligibility for DMTs has expired.

Ensuring health care system readiness for the adoption of recent and forthcoming biomedical innovations in early Alzheimer's care requires the removal of system barriers that hinder PCPs from making timely identification of treatment‐eligible individuals commensurate with the prevalence of early AD. Drawing upon the work of a multistakeholder consortium, we aim to (1) identify barriers to PCPs in the United States playing a larger role in the detection, diagnosis, treatment, and monitoring of early AD; (2) propose solutions to these barriers; and (3) encourage evolution of the U.S. healthcare system to ensure readiness to adapt to the rapidly progressing scientific landscape and meet the growing needs of people affected by the early stages of AD.

## 3 INCREASING THE ROLE OF PRIMARY CARE

One approach to addressing this insufficient infrastructure is to equip primary care teams with the necessary tools and education to detect, diagnose, treat, and monitor patients with AD. Primary care is traditionally defined by its core functions, often termed the “4Cs,” of first contact, comprehensiveness, coordination, and continuity.^24^ It is also the most common point of care in the U.S. and is associated with improved health outcomes, fewer hospitalizations, higher quality services, greater equity, better population health, and lower costs.^25^ One simulation study projected that increasing primary care's role in AD could substantially improve diagnosis and treatment with DMTs, reducing the burden on an already strained neurology and geriatric specialist workforce.^12^

Many other chronic conditions, once managed by specialists, are now routinely managed in primary care. These include diabetes, hypertension, asthma, chronic obstructive pulmonary disease, chronic kidney disease, depression, and anxiety. Key drivers of this shift from specialty care to primary care—consistent with AD care today—include the availability of medications to manage these conditions and the challenges of multimorbidity, where generalist care is more practical and improves coordination of care.^26^

Although PCPs are on the front lines of patient care, they face many well‐documented challenges in detecting, diagnosing, and managing AD. These include knowledge gaps, inadequate training, and lack of confidence in diagnosis; severe time constraints; resource limitations; and insufficient awareness of community services.^27^ As a result, primary care practices do not routinely perform or document cognitive assessments.^28^ One major policy effort to promote timely diagnosis of AD in primary care is the Medicare Annual Wellness Visit (AWV), which includes a required brief cognitive assessment. However, even though many Medicare beneficiaries receive an AWV, PCPs frequently do not use validated cognitive assessment instruments, and even when results suggest cognitive impairment, they often fail to initiate any clinical follow‐up or relay a possible diagnosis of AD to their patients.^29^, ^30^

Although beyond the scope of this paper, shifting AD care toward greater primary involvement must also address the existing workforce crisis, including shortages of PCPs and other supporting clinicians, increased administrative burden, widespread burnout, and inadequate reimbursement.^31^

## 4 ADDRESSING STRUCTURAL BARRIERS TO EMPOWER AN INCREASED ROLE FOR PRIMARY CARE

In developing solutions to reduce system barriers in AD detection, diagnosis, treatment, and monitoring over the next 5 to 10 years, we made several key assumptions. First, we assumed that detection would become more efficient and accurate through greater use of digital assessment tools and highly accurate blood‐based biomarker (BBM) tests for both people with and without symptoms. Second, we assume that subcutaneous DMTs will be FDA‐approved and available for treatment initiation and maintenance, thereby reducing the barriers and additional costs associated with infused DMTs. Third, we assume that treatment side effects will be safely managed either through improved drug delivery techniques (e.g., brain shuttle technology), refinement of dosing regimens, adjunct medications (e.g., corticosteroids given before amyloid‐targeting therapy), or the FDA approval of DMTs with a better side effect profile. The main side effect of current amyloid‐targeting therapies is the development of amyloid‐related imaging abnormalities (ARIA), which can involve brain swelling and bleeding. Given these assumptions, the Consortium focused on the remaining barriers and potential solutions that would enable PCPs to deliver a greater share of AD care.

### 4.1 Knowledge gaps

First, knowledge gaps must be addressed through education and training for PCPs and their teams. PCPs often report lacking the knowledge and tools needed to evaluate cognitive concerns.^18^ To address this gap, education is needed for entire primary care teams—including physicians, physician assistants (PAs), nurse practitioners (NPs), care navigators, and others—on how to identify potential cognitive issues and objective overviews on the efficacy of available treatments that can slow disease progression. Such education could increase the likelihood that clinicians engage in brain health discussions and give them greater confidence that treatments will improve patients’ lives. Education programs should be tailored to the needs and schedules of PCPs and their teams. One qualitative study found that PCPs often prefer brief and flexible training programs that leverage technology such as recorded videos or live webinars offered during their lunch hour. When these programs provide continuing medical education (CME) credits, there is an additional incentive to enroll in the training.^32^ Targeted, web‐based CME participation is associated with 1.6 times more AD diagnoses by PCPs when compared to similar PCPs who did not participate.^33^ Other approaches, such as the Cognition in Primary Care intervention at the University of Washington health system, found that providing integrated education combined with in‐exam room cognitive assessment tools led to significant increases in both cognitive assessments (from 2.8 to 19.8 per month per PCP) and new AD diagnoses (from 6.2 to 14.6 per month per PCP) by PCPs.^34^

PCPs also need education designed to help them choose, administer, and document validated cognitive assessments, such as the Montreal Cognitive Assessment (MoCA) or the St. Louis University Mental Status Exam (SLUMS). Such training should include choosing culturally appropriate tools as well as guidance on ordering and interpreting BBM tests. Pilot programs that include education and decision support via electronic health record (EHR)‐based checklists have shown promise.

Practice guidelines are needed for AD detection, diagnosis, treatment, and monitoring. Clinical pathways—similar to those used in cancer care—could give primary care teams a structured yet flexible process to navigate shared decision‐making for this complex condition. However, unlike cancer, AD guidelines have typically come from patient advocacy groups rather than from professional societies. If that trend continues, professional societies should formally acknowledge and endorse guidelines developed by patient advocacy groups. As a first step, guidelines should be developed for cognitive assessment, including specific recommendations for follow‐up care when indicated by cognitive assessment results. These guidelines could help improve the current estimates that only 17% to 35% of patients who screen positive on cognitive assessments receive more detailed cognitive screening, a referral for neuropsychological testing, neuroimaging orders placed, or a referral to a dementia subspecialty clinic from their PCP, often because the physician is unsure what to do next.^29^

Additionally, the use of support systems such as Project ECHO (Extension for Community Healthcare Outcomes) and the Institute for Healthcare Improvement (IHI) Age Friendly Health Systems initiative can provide access to clinical education and toolkits and direct PCP access to memory specialists who can provide additional guidance when questions arise.^35^, ^36^

### 4.2 New technologies

Second, new technologies can help identify higher‐risk individuals and reduce the workload on primary care teams.

Increased use of artificial intelligence (AI) ‐driven risk stratification could help prioritize patients for biomarker testing or cognitive assessment, thus optimizing resources by focusing on those at higher risk of developing AD. These tools integrate clinical, genetic, laboratory, imaging, and proteomic data, as well as information from passive digital markers such as wearable sensors and device cameras. Machine learning algorithms are applied to predict risk and guide AD testing with validated instruments. Such methods have demonstrated the ability to detect AD pathology or predict dementia diagnosis years before symptoms appear.^37^, ^38^ Running in the background, these tools can detect subtle changes and trigger additional testing for specific individuals. However, as AI tools become more widely deployed, it is critical that they be developed and validated using diverse datasets reflecting the populations disproportionately affected by AD—particularly women, Blacks, Hispanics, and people with Down syndrome or other intellectual and developmental disabilities (IDD). This will help prevent algorithmic bias from exacerbating existing disparities in early detection and access to treatment. Standards and dedicated funding are also needed to ensure safe review and secure storage of these data. New tools can create additional barriers to implementation that need to be addressed, particularly in the additional time and resource burdens placed on PCPs should they need to store and review data from these technologies. Policies that encourage healthcare organizations to collect and house these data, such as a Center for Medicare and Medicaid Innovation (CMMI) demonstration pilot or incentives to Accountable Care Organizations, would reduce some of these burdens on PCPs.

Additionally, routine use of digital cognitive assessments (DCAs) should be increased. Advanced neuropsychological testing tools, such as DCAs, have not yet seen widespread adoption. Currently, DCAs are used primarily in clinical trial recruitment, demonstration projects, or specialized Alzheimer's care programs at academic centers. DCAs provide scalable, efficient, and sensitive detection of cognitive impairment, which is often missed by traditional pen‐and‐paper assessments.^39^ DCAs can be integrated into routine visits and even be done online prior to a patient visit, providing a longitudinal view of a patient's cognitive health and prompting PCPs to initiate diagnostic workups sooner. A study conducted at the University of California at San Francisco (UCSF) Lakeshore Family Medicine Clinic with 23 PCPs and 11 medical assistants (MAs) demonstrated the feasibility and impact of DCAs, with over 95% of PCPs adopting use of the tool and leading to PCP diagnosis rates 1.72 times higher for patient visits among those aged 65 and older and referrals to specialty clinics increasing 1.66 times after implementation of the DCA.^40^ Use of DCAs has been shown to increase PCP confidence in their clinical abilities—an important factor in feeling prepared to diagnose and manage patients with AD.^41^ When paired with highly accurate BBM tests, DCAs have been shown to predict progression of AD, even in currently cognitively unimpaired individuals.^42^ Accelerating the use of DCAs could be achieved if EHR companies integrated them into EHRs, thus reducing the cost and time burden on primary care practices to integrate them individually. Such integration is also in alignment with the final rule of the Department of Health and Human Services’ Electronic Health Record Reporting Program provision of the 21st Century Cures Act.

At present, AD diagnosis relies primarily on clinical symptoms, supplemented by neuropsychological tests, advanced imaging, primarily positron emission tomography (PET), and—when available—BBM tests. The advent of BBM tests, which approximate the presence and burden of amyloid plaques in the brain, may make early diagnosis easier, faster, and less expensive for both PCPs and patients compared to cerebrospinal fluid (CSF) studies or PET scans. BBMs, similar to PET and CSF biomarkers, are not diagnostic on their own. However, the evidence supports BBM testing as a reliable component of the AD diagnostic workup when interpreted in the context of clinical presentation and other available data. One study found that the PCPs’ diagnostic accuracy for AD pathology after a clinical evaluation of patients with MCI or dementia due to AD increased from 61% to 91% with the addition of BBMs.^42^ The Alzheimer's Association recently published clinical guidelines on the use of BBMs in specialized care settings (which may include primary care physicians, NPs, and PAs).^43^

Expanding the use of BBMs could streamline the diagnostic process and help identify patients with probable AD without the resource‐intensive steps currently required. The recent availability of standardized, high‐accuracy biomarker tests can enable PCPs to generate the objective evidence required to satisfy prior authorization and “medical necessity” criteria, thereby unclogging the administrative bottleneck that currently restricts access to these covered therapies. To reach this goal, PCPs need training on the proper use of these tests, understanding which tests to order, interpreting results, and communicating findings to patients as well as training on.

Establishing broad patient access for these blood tests is critical to their widespread adoption in primary care, both now for AD patients with symptoms and in the future when they are validated for patients with AD pathology but without apparent symptoms. For AD patients with pathology but without symptoms, Centers for Medicare and Medicaid Services (CMS) and other payers can create coverage for biomarker testing as part of a cognition risk assessment for higher‐risk individuals based on such factors as age, family history, and modifiable factors such as obesity and high blood pressure. Currently, state biomarker laws in over 20 states mandate that health insurance plans, including many Medicaid and state employee plans, cover biomarker testing for diagnosis, treatment, and management for cancer, AD, and other diseases if such tests are FDA‐cleared. This type of coverage is possible for Medicare plans as well. CMS recently demonstrated authority over other screening payment coverage decisions for risk assessments for atherosclerotic cardiovascular disease^44^ and social determinants of health.^45^ The recent bipartisan Alzheimer's Screening and Prevention (ASAP) Act, if passed, would encourage CMS to develop criteria for the use of BBMs as assessment instruments for people without AD symptoms to provide clarity for healthcare professionals and help promote consistent coverage policies by private payers.^46^

Diagnostic testing with PET scans, CSF analysis, or BBMs helps avoid the false positives seen with sole use of traditional cognitive assessments and has demonstrated similarly robust clinical validity across diverse populations, and clinical decision‐making based on BBMs has been shown not to be affected by a patient's race or ethnicity.^47^, ^48^ AD biomarker testing can also be preceded by or combined with blood tests for common reversible conditions (such as vitamin B12 deficiency or hypothyroidism) that can present as memory issues or exacerbate cognitive symptoms.

### 4.3 Reimbursement policies

Third, reimbursement policies must be aligned to support detection, diagnosis, treatment, and monitoring in primary care.

Ensuring appropriate reimbursement is essential to adequately compensate PCPs for the work of AD detection and diagnosis. Current Current Procedural Terminology (CPT) codes and Medicare Physician Fee Schedule rules impose time and provider‐type requirements that few PCPs can meet. PCPs need codes that allow more appropriate billing for cognitive assessments, interpretation of assessment results, and care planning. The CMS 2026 Physician Fee Schedule introduced a new quality improvement activity aimed at improving detection of cognitive impairment in primary care, but further action is needed. CMS's Quality Payment Program could tie incentive payments to specific requirements primary care clinicians would have to meet, such as established baseline detection rates and increased Medicare AWV cognitive assessment uptake using validated cognitive assessments for patients over the age of 65. CMS could also incentivize earlier AD detection and diagnosis by adding a risk‐adjusted payment for MCI in Medicare Advantage plans. Based on the experience after CMS added a risk adjustment for dementia, this step could improve diagnosis rates.

New payment models are needed to support care coordination, such as incorporating patient navigators and enabling team‐based primary care. Beyond expanding existing billing codes, one approach would be for CMS to extend key concepts of the GUIDE (Guiding an Improved Dementia Experience)^49^ model to include MCI as well as coordination of DMT initiation and monitoring in an Alzheimer's‐specific per‐beneficiary‐per‐month (PBPM) payment for primary care along with the current risk‐adjusted monthly payments to practices that deliver documented care plans, engage care partners, and connect them with community‐based supports, and provide navigation services.

### 4.4 Capacity constraints

PCP capacity constraint pressures can be lessened by optimizing clinical workflows, implementing team‐based care, and incorporating patient navigators. Team‐based models that utilize PAs, and NPs, as well as non‐clinicians, to handle parts of the assessment and diagnostic process can increase capacity and reduce the time burden on PCPs. For example, trained staff (such as MAs) can administer, score, and even interpret brief cognitive assessments.

The CMS’ GUIDE model could be broadened to include additional providers (beyond dementia experts and care navigators) and patients at earlier stages of AD. At present, eligibility is restricted to those with confirmed dementia diagnoses and excludes individuals with MCI or unimpaired individuals with AD pathology who could benefit from closer cognitive monitoring and more aggressive management of modifiable risk factors such as hypertension. Additionally, support programs such as Project ECHO can connect primary care teams with memory specialists and toolkits, extending the care team and providing guidance when questions arise.^35^

Telehealth services are also an increasingly useful supplement or adjunct to the capabilities of PCPs, particularly primary care practices that are not integrated with or within provider networks that include specialty services such as neurology or geriatric psychiatry. Evidence suggests that virtual specialist consultations can expedite accurate interpretation of cognitive assessment and other elements of neuropsychological consulting and that many elements of neuropsychological testing can be administered directly over telehealth platforms.^50^

### 4.5 Equity and patient‐centeredness

All approaches must be implemented with a focus on equity and patient‐centeredness, tailoring solutions to different patient populations and individual circumstances. Minoritized populations, people with Down syndrome or other IDDs, those living in rural or medically underserved areas, and individuals facing socioeconomic disadvantages experience heightened and persistent access barriers to AD care at all stages. They are less likely to be assessed for cognitive impairment or diagnosed (especially in early stages of AD), and they experience longer delays as well as financial, geographic, and other barriers to treatment when eligible.^13^ Recommended solutions should be tailored to the specific population being served and the reality of the care setting in which they receive (or should receive) care. The overall goal should be to provide culturally appropriate, person‐centered care in the communities where patients and their care partners live.

Solutions designed to support primary care must explicitly address the experiences of women as both patients and caregivers. Women represent two‐thirds of people in the United States living with AD and provide the majority of unpaid caregiving—creating a compounded impact on women's health, economic security, and quality of life. Any proposed solution should consider sex‐specific risk factors, the intersection of AD with other conditions that disproportionately affect women, and the financial vulnerabilities created when working‐aged women develop AD or assume caregiving roles.

Another equity concern is the potential discrimination against those diagnosed with AD—particularly individuals who are unimpaired or at the MCI stage and still working. Policymakers may need to regulate the disclosure of biomarker test results and cognitive assessment outcomes—similar to how genetic information is protected—to shield individuals from discrimination in employment, long‐term care insurance, driving privileges, and housing.^51^

Additionally, patient‐centeredness also requires that patients participate fully in shared decision‐making and are aware of both the benefits and risks of treatment (and nontreatment) options. Without systems structured toward making access for all those who desire treatment viable, disparities will continue.

## 5 STRUCTURAL PROBLEMS BENEFIT FROM A SYSTEM APPROACH

Finding scalable solutions to reduce system bottlenecks in early AD detection, diagnosis, treatment, and monitoring is an urgent priority. If these challenges are not addressed, the human, economic, and health system costs of delay will continue to mount as the number of AD patients grows in the coming years.

For instance, we now know that nearly half of dementia cases may be delayed or prevented, and in some cases, MCI can be stabilized or even reversed with lifestyle and medication management.^52^, ^53^ The release of the U.S. POINTER study results in July 2025 provided definitive evidence that structured, multidomain lifestyle interventions can significantly improve cognition in older adults at risk for decline.^54^ These findings, which replicate and expand upon the FINGER trial model in a diverse U.S. population, confirm that a “brain health recipe” of physical exercise, nutritional guidance, and cognitive challenge is a scalable, effective intervention that falls squarely within the scope of primary care. This is further reinforced by the 2024 update to the *Lancet Commission on Dementia Prevention, Intervention, and Care*, which identified that nearly 45% of dementia cases are attributable to 14 modifiable risk factors, including the newly added factors of untreated vision loss and high low‐density lipoprotein (LDL) cholesterol.^55^ Managing these risks—along with hypertension, diabetes, and hearing loss—constitutes a “primary care mandate” that enables PCPs to take immediate, cost‐effective action to build cognitive reserves. By integrating the “POINTER protocol” into routine care, the healthcare system can proactively address brain health decades before pathology manifests, offering a powerful tool to stabilize MCI and reduce the aggregate burden of disease alongside DMTs.

However, none of this is possible without a discussion about brain health in primary care that is part of routine care throughout the lifespan. Incentives and policies are needed to make brain health a regular part of primary care visits. Several of these have been outlined by a consensus recommendations paper following an UsAgainstAlzheimer's risk reduction work group.^52^

Expanding primary care's role presents both an opportunity and a challenge. The opportunity is that people with AD can enjoy a higher quality of life and remain independent longer, with reduced spending on residential care, emergency room visits, and disease‐related complications. The challenge is to find solutions that work for all stakeholders.

It is particularly important to consider how specific solutions might impact the rest of the AD care ecosystem. Often, solutions implemented to address one problem have unintended consequences in other areas of care. For example, increasing cognitive assessments without addressing downstream diagnostic bottlenecks can leave patients worried but unable to receive a definitive diagnosis or treatment.^28^

Point solutions, while easier to implement, will not be sufficient to create the level of change needed. Given the scale and urgency of the challenge, multiple coordinated solutions must be implemented simultaneously. In short, the system must evolve. Solutions must be coordinated, and outcomes from pilot programs should be measured and shared using the lessons learned to improve future programs. PCPs need timely, efficient, and practical education and tools to meet the needs of their patients, as well as other resources, including appropriate reimbursement, which will allow them to staff primary care practices adequately.

To achieve the goal of coordinated and efficient system change, various stakeholders, including relevant government agencies, public and private insurers, system administrators, health care professional societies, biopharmaceutical companies, EHR platforms, and patient advocacy groups, could work together to make system‐wide improvements and align incentives toward improvements that benefit current and future patients without unduly burdening a single stakeholder group.

## 6 SUMMARY

AD is a growing public health crisis that the health care system must address. It is the sixth leading cause of death in the United States and among the most expensive and burdensome conditions—costing the health care system $384 billion annually, not including the $413 billion in care provided by 12 million unpaid care partners.^5^

Delaying the progression of AD benefits not only patients and their families but also payers, the economy, and society at large. However, an ill‐prepared and inefficient system often leaves AD unchecked, leading to poorer quality care, reduced quality of life for patients and families, greater institutionalization, widening inequities in care, and an increased economic burden.

We now have pharmacological treatments that have shown efficacy for early symptomatic AD that can change this trajectory, with more on the way. These therapies for AD currently sit in the gap between past practices and future possibilities. When the health care system takes years to deliver an FDA‐approved product to its intended patients, it undermines health outcomes, prolongs health disparities, wastes valuable resources, and threatens the sustainability of both biomedical innovation and care delivery. The solution lies in recognizing that such gaps in “biomedical health efficiency” can be bridged only when stakeholders work collaboratively—rather than in parallel—on system redesign.^56^

## CONFLICT OF INTEREST STATEMENT

S.E.P. and G.H. are full‐time employees of Tufts Medical Center, which receives support from the NEWDIGS consortium under the Tufts Center for Biomedical System Design (not specific to the present manuscript). I.K. is the Executive Director of the LEAD Coalition, the coalition's sole source of revenue is from member organization dues (30% of members involved in drug, device, or clinical services; 70% are patient advocacy or similar groups), and has received small honorariums from Quanterly/Lucent for roundtable participation, Digital Medicines Society for workgroup participation, NYU Public Health Center of Excellence on Dementia Detection and Diagnosis for advisory committee participation, and the National Association of Managed Care Physicians for an educational presentation, and a small consulting fee from Archo for survey question design on promoting brain health. J.K.W. is an employee and minor shareholder of Eli Lilly & Company and an unpaid board member of the Indiana Biblical Counseling Center. J.W. receives compensation from the American Academy of Family Physicians and USAgainstAlzheimers as a consultant, has received support to attend meetings from the American Academy of Family Physicians and the American College of OB‐GYN, serves on the Women's Preventive Services Initiative Advisory Panel and the Rural Health Information Advisory Board. M.B. is an employee of the Alzheimer's Association and serves on the National Hypertension Control Roundtable Steering Committee, Milken Institute Alliance to Improve Dementia Care Steering Committee, Dementia Friendly America Steering Committee, and Korea National Institute of Dementia International Advisory Board, and has received NIA Summer Research Institute grant, NIH subgrant from University of Southern California – Economics of Alzheimer's, CDC BOLD Center of Excellence on Dementia Risk Reduction, and a CDC subgrant from the University of Minnesota BOLD Center of Excellence on Dementia Caregiving, as well as an honorarium for service on the International Advisory Board for the Korea National Institute of Dementia (paid as a donation to Alzheimer's Association), and complimentary registration to the Milken Institute Future of Health Summit in 2024 and 2025. S.D. is an employee of AHIP. M.F. is an employee of Highmark Health. T.H. receives compensation from Tufts Medical Center as a project consultant for the NEWDIGS Consortium, in the past 36 months was an employee of NEHI Inc which received project sponsorships from Eisai Inc., Eli Lilly & Company, and Genentech, Inc (paid directly to NEHI). A.J. is a full‐time employee of Linus Health, receives stock options and meeting support from Linus Health, has pending patents on digital assessment of CNS functionality, digital biomarkers of cognitive, functional, and amyloid‐positivity status, and machine learning‐enabled clinical decision support. M.M. receives compensation and stock options from C2N Diagnostics, LLC as a consultant. C.R. is an employee of Tufts Medical Center. Author disclosures are available in the Supporting Information.

## CONSENT STATEMENT

Consent (i.e., all human subjects [provided consent) was not applicable.

## Supporting information



Supporting Information
